# Proinflammatory Effects of Ubiquitin-Specific Protease 5 (USP5) in Rheumatoid Arthritis Fibroblast-Like Synoviocytes

**DOI:** 10.1155/2020/8295149

**Published:** 2020-03-09

**Authors:** Xiao-Bo Luo, Jian-Cheng Xi, Zhen Liu, Yu Long, Li-tao Li, Zhan-Peng Luo, Dao-Hong Liu

**Affiliations:** Department of Orthopedic, Eighth Medical Center of PLA General Hospital, Beijing 100091, China

## Abstract

Rheumatoid arthritis (RA) is a worldwide chronic autoimmune inflammatory disease which is affecting approximately 1% of the total population. It is characterized by abnormal proliferation of fibroblast-like synoviocytes (FLS) and increased production of proinflammatory cytokines. In the current study, we were aiming to investigate the role of ubiquitin-specific protease 5 (USP5) in the inflammatory process in RA-FLS. Expression of USP5 was found upregulated in RA-FLS compared with that in osteoarthritis- (OA-) FLS, and IL-1*β* stimulation increased USP5 expression in a time-dependent manner. Furthermore, we found that USP5 overexpression significantly aggravated proinflammatory cytokine production and related nuclear factor *κ*B (NF-*κ*B) signaling activation. Consistently, silencing of USP5 decreased the release of cytokines and inhibited the activation of NF-*κ*B. In addition, USP5 was found to interact with tumor necrosis factor receptor-associated factor 6 (TRAF6) and remove its K48-linked polyubiquitination chains therefore stabilizing TRAF6. Our data showed that a USP5-positive cell regulates inflammatory processes in RA-FLS and suggested USP5 as a potential target for RA treatment.

## 1. Introduction

Rheumatoid arthritis (RA) is a chronic autoimmune inflammatory disease and is characterized by the inflammation of joints, subsequent destruction of cartilage, and erosion of the bone [1, 2]. At present, the main clinical treatment strategy of RA is drug therapy, including immunosuppressive drugs and biological agents. However, these therapies induce a general drug resistance that increases the risk of infectious diseases and cancer [3]. Therefore, it is of great significance to explore the mechanism of RA and identify new therapeutic targets.

Fibroblast-like synoviocytes (FLS) are a special type of mesenchymal-derived cells lining the layer of normal synovial tissues [4]. In RA patients, FLS surprisingly express some phenotypes similar to tumor cells that are described as unregulated proliferation, resistance to apoptosis, and insensitivity to contact inhibition [5]. In addition, RA-FLS contribute to the production of proinflammatory cytokines and matrix metalloproteinases (MMPs) that lead to cartilage destruction [6]. Recently, the central role of FLS in the progression of RA makes FLS considered for RA interventions in a therapeutic manner [7–9].

Ubiquitination is an important posttranslational modification to regulate various cellular processes [9]. Deubiquitination, the reverse step of ubiquitination, is mediated by deubiquitinating enzymes (DUBs) that specifically cleave ubiquitin from ubiquitin-conjugated protein substrates [10]. Ubiquitin-specific protease 5 (USP5) belongs to the ubiquitin-specific protease (USP) family, and it has been identified as a tumor promoter in several types of human cancer. Increased ubiquitin-specific protease 5 (USP5) has been associated with tumorigenesis of malignancy including pancreatic cancer, melanoma, non-small-cell lung cancer, and hepatocellular carcinoma [11–13]. Recent study also showed that USP5 is essential for the production of TNF-*α* in mucosal mast cells [14]. However, the effect of USP5 in RA still remains largely unknown.

In the current study, we were aiming to investigate the effect of USP5 on inflammatory responses in IL-1*β*-stimulated RA-FLS. We found that the expression of USP5 was increased in RA-FLS compared with that in OA-FLS. Furthermore, there was a positive correlation between USP5 expression and proinflammatory cytokine production. USP5 was found to aggravate NF-*κ*B activation, and USP5 was observed to interact with TRAF6, leading to the K48-linked deubiquitination of TRAF6. These data suggested USP5 as a positive regulator of IL-1*β*-induced inflammatory responses, and it provide a new potential target for the research and development of RA.

## 2. Material and Methods

### 2.1. FLS and Cell Culture

FLS were isolated from the synovial tissues of RA or OA patients as previously reported [9]. The tissues were collected from 8 RA patients and 8 osteoarthritis (OA) patients from the Department of Orthopedic of Eighth Medical Center of PLA General Hospital; RA patients fulfilled the 2010 ACR/European League Against Rheumatism (EULAR) classification criteria for RA. Informed consent was obtained from all patients, and the study protocol was approved by the Ethics Committee of our hospital. The FLS cells were grown in DMEM (Gibco-Invitrogen Corp., Rockville, MD) and supplemented with penicillin, streptomycin, and 10% fetal bovine serum (FBS) and incubated at 37°C in a 5% CO_2_ condition. FLS cells from passages three to five were used.

### 2.2. Quantitative PCR Analysis

Total RNA was extracted from synovial tissues or FLS cells using a TRIzol reagent (Invitrogen, USA), and cDNA was then synthesized with a PrimeScript RT Reagent Kit (TaKaRa, Japan) according to the manufacturer's protocol. 500 ng cDNA was used for quantitative PCR with a QuantiTect™ SYBR Green PCR kit (TaKaRa, Otsu, Japan). Cycling conditions were 1 cycle (95°C, 5 min) and 40 cycles (95°C, 15 sec; 55°C, 30 sec; and 72°C, 30 sec); the 2^-*ΔΔ*CT^ method was used to evaluate the relative quantities of each amplified product in the samples [15]. Primer sequences used are shown in Table 1.

### 2.3. Lentivirus and Plasmid

All of the lentivirus and plasmids were produced by MDL Technology Company (MDL Biotech, Beijing, China). Lentivirus containing vector plasmid (control) and overexpression plasmid of USP5 was constructed by using a pLenti6/V5 vector (Life Technologies); lentivirus containing negative control plasmid (sh-NC) and shRNA plasmid of USP5 (sh-USP5) was constructed by using a pLKO.1 vector (Sigma-Aldrich). Lentivirus was obtained and enriched according to the manufacturer's instruction. FLS cells were infected at 50 MOI of lentivirus, and jetPEI reagents (Polyplus-transfection) were used for the transfection of Myc-USP5, Flag-TRAF6, and HA-ub plasmids.

### 2.4. Western Blot Analysis, Immunoprecipitation, and Ubiquitination Assay

The experiments were performed according to the previous report [9]. Proteins were quantified using a BCA protein assay kit (Thermo Scientific, Waltham, MA), and 25 *μ*g proteins were separated using 10% SDS-PAGE and transferred to a PVDF membrane (Millipore). The antibodies for USP5 (1 : 500, #ab155993), TRAF6 (1 : 500, #ab33915), p65 (1 : 500, #ab16502), phos-p65 (1 : 500, #ab76302), I*κ*B*α* (1 : 500, #ab32518), phos-I*κ*B*α* (1 : 500, #ab133462), *β*-actin (1 : 1000, #ab179467), Myc (1: 1000, #ab32), Flag (1 : 1000, #ab49763), and K48-ub (1 : 500, #ab140601) were all bought from Abcam (Abcam, Cambridge, USA).

### 2.5. ELISA

The levels of interleukin-6 (IL-6), tumor necrosis factor-alpha (TNF-*α*), and matrix metalloproteinase 1 (MMP1) were measured by ELISA kits (R&D Systems) according to the manufacturer's instructions.

### 2.6. Dual-Luciferase Reporter Gene Assays

The experiments were performed as described in [16]. Luciferase activity of the NF-*κ*B promoter was evaluated by the Dual-Luciferase Reporter Assay System (Promega, Madison, USA) and detected by a spectraMax M5 reader (Molecular Devices, California, USA).

### 2.7. Statistical Analysis

All data are presented as the mean ± S.D. of three independent experiments. Statistical significance was determined using two-tailed Student's *t*-test to compare the two groups. One-way ANOVA was performed to compare three or more. If the analysis of variance was significant, the Newman-Keuls test was used to compare each group. The correlations of the expressions of USP5 and TRAF6 were established by Pearson's correlation analysis. *p* values < 0.05 are considered statistically significant.

## 3. Results

### 3.1. USP5 Expression Was Increased in RA-FLS Compared with OA-FLS

**Figure 1:**
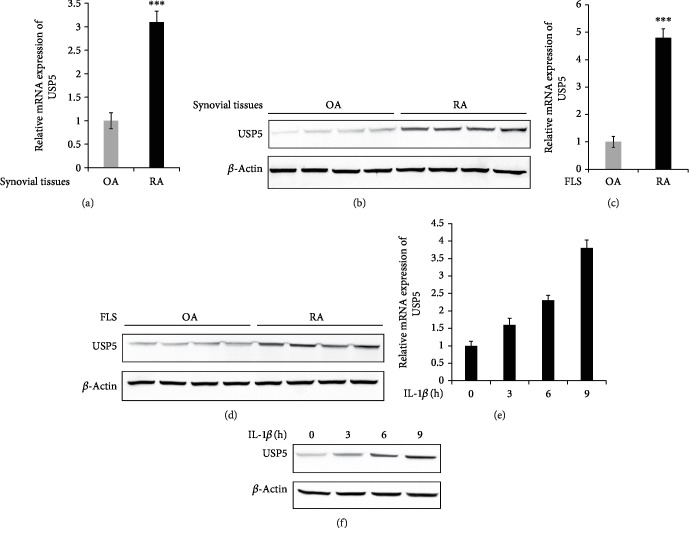
USP5 expression was increased in RA-FLS compared with OA-FLS. mRNA (a) and protein (b) levels of USP5 in synovial tissues from 8 RA patients and 8 OA patients. mRNA (c) and protein (d) levels of USP5 in FLS from 8 RA patients and 8 OA patients. mRNA (e) and protein (f) levels of USP5 in RA-FLS after IL-1*β* treatment (10 ng/ml) for the indicated time. ^∗∗∗^*p* < 0.001.

To explore the effect of USP5 on RA, we examined the expression of USP5 at first. As shown in Figures 1(a) and 1(b), both mRNA and protein levels of USP5 were markedly upregulated in synovial tissues of RA patients compared with OA synovial tissues. Furthermore, we detected the expression of USP5 in FLS cells from RA and OA patients. Consistently, the mRNA and protein levels of USP5 were also increased in RA-FLS compared with OA-FLS (Figures 1(c) and 1(d)). These findings suggested us that USP5 expression was increased in RA patients, which indicated that USP5 may participate in the development of RA. Interleukin-1 beta (IL-1*β*) is the most common proinflammatory stimuli involved in RA pathogenesis; therefore, we stimulated RA-FLS with IL-1*β* to investigate the expression of USP5 in response to proinflammatory cytokines. We found both the mRNA and protein levels of USP5 were increased after IL-1*β* treatment in a time-dependent manner in RA-FLS (Figures 1(e) and 1(f)).

### 3.2. Overexpression and Silence of USP5 by Using Lentivirus

**Figure 2:**
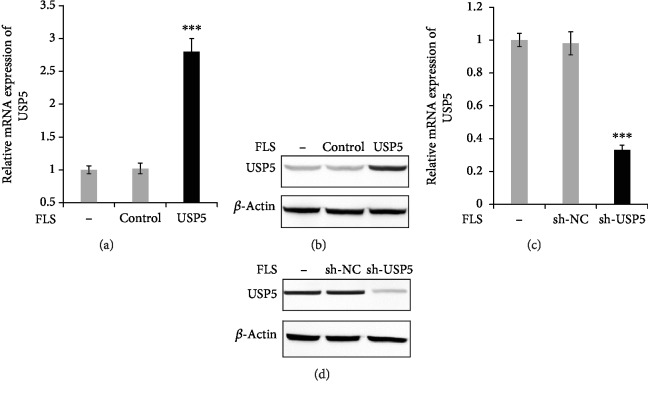
Overexpression and silencing of USP5 by using lentivirus. (a, b) RA-FLS were infected with lentivirus containing empty plasmid (control group) or USP5 expression plasmid (USP5 group) for 48 hours; the efficiency was confirmed in both mRNA (a) and protein (b) levels. RA-FLS were infected with lentivirus containing negative control shRNA plasmid (sh-NC group) or USP5 shRNA plasmid (sh-USP5) for 48 hours; the efficiency was confirmed in both mRNA (c) and protein (d) levels. ^∗∗∗^*p* < 0.001.

For the further research, we used lentivirus to overexpress or silence USP5 expression. The RA-FLS cells were infected with lentivirus containing USP5 expression plasmid, and the efficiency was confirmed in both mRNA (Figure 2(a)) and protein levels (Figure 2(b)). Furthermore, we constructed lentivirus containing shRNA plasmid of USP5, and the silence efficiency is shown in Figures 2(c) and 2(d).

### 3.3. USP5 Aggravated Proinflammatory Cytokine Production in IL-1*β*-Treated RA-FLS

**Figure 3:**
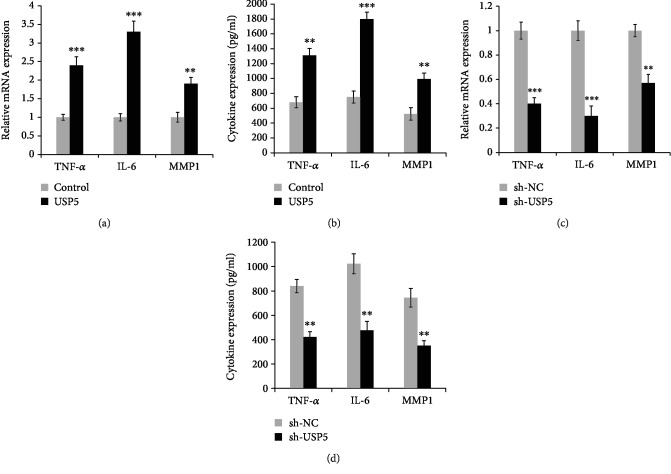
USP5 aggravated proinflammatory cytokine production in IL-1*β*-treated RA-FLS. mRNA (a) or secreted protein (b) levels of TNF-*α*, IL-6, and MMP1 in USP5-overexpressed RA-FLS after IL-1*β* (10 ng/ml) treatment for 9 hours. mRNA (c) or secreted protein (d) levels of TNF-*α*, IL-6, and MMP1, in USP5-silenced RA-FLS after IL-1*β* (10 ng/ml) treatment for 9 hours. ^∗∗^*p* < 0.01, ^∗∗∗^*p* < 0.001.

Proinflammatory cytokine production in FLS was critical for the pathogenesis of RA. Thus, we detected the effect of USP5 on proinflammatory cytokine production in IL-1*β*-treated RA-FLS. We found USP5 markedly promoted the production of cytokines such as tumor necrosis factor-alpha (TNF-*α*), interleukin-6 (IL-6), and matrix metalloproteinase 1 (MMP1) (Figure 3(a)). In addition, the ELISA data suggested that the secreted protein levels of TNF-*α*, IL-6, and MMP1 were also enhanced in USP5-overexpressed RA-FLS after IL-1*β* treatment (Figure 3(b)). Consistently, both mRNA and protein levels of proinflammatory cytokines were also decreased in USP5-silenced RA-FLS cells (Figures 3(c) and 3(d)).

### 3.4. USP5 Promoted NF-*κ*B Signaling Activation in IL-1*β*-Treated RA-FLS

**Figure 4:**
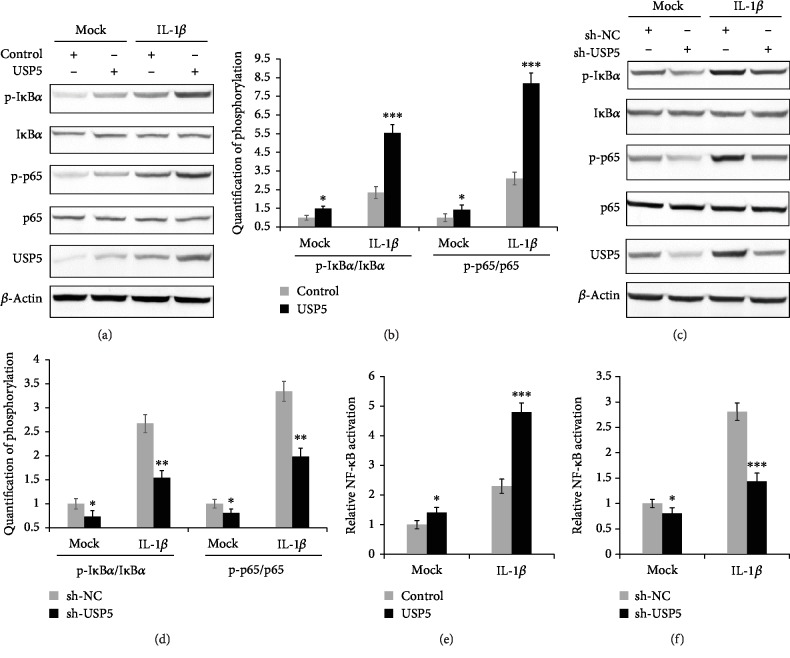
USP5 promoted NF-*κ*B signaling activation in IL-1*β*-treated RA-FLS. (a) Phosphorylation levels of I*κ*B*α* and p65 in USP5-overexpressed RA-FLS after IL-1*β* (10 ng/ml) treatment for 9 hours. (b) Quantification of phosphorylation of I*κ*B*α* and p65 in (a). (c) Phosphorylation levels of I*κ*B*α* and p65 in USP5-silenced RA-FLS after IL-1*β* (10 ng/ml) treatment for 9 hours. (d) Quantification of phosphorylation of I*κ*B*α* and p65 in (c). (e, f) After infection with lentivirus containing USP5 expression plasmid (e) or USP5 shRNA plasmid (f) for 48 hours, RA-FLS were transfected with NF-*κ*B reporter plasmid and phRL-TK plasmid (internal control) for 24 hours, followed by the stimulation with IL-1*β* (10 ng/ml) for 9 hours; the activation of the NF-*κ*B promoter was measured by a dual-luciferase reporter gene assay. ^∗^*p* < 0.05, ^∗∗^*p* < 0.01, and ^∗∗∗^*p* < 0.001.

Recently, many studies showed that NF-*κ*B signaling is crucial for the RA process, especially in the proinflammatory cytokine production in FLS [4, 9, 16]. Therefore, we detected the effect of USP5 on IL-1*β*-induced NF-*κ*B activation. As shown in Figures 4(a) and 4(b), we found that the phosphorylation of I*κ*B*α* and p65 was both aggravated in USP5-overexpressed RA-FLS cells. In addition, knockdown of the expression of USP5 was found to suppress the phosphorylation of I*κ*B*α* and p65, which indicated that USP5 suppressed the activation of NF-*κ*B (Figures 4(c) and 4(d)). Moreover, we used a dual-luciferase reporter assay to investigate the activation of the NF-*κ*B promoter, and we observed that USP5 significantly promotes NF-*κ*B activation (Figures 4(e) and 4(f)). During these experiments, we also observed that USP5 could slightly increase NF-*κ*B activation without IL-1*β*; however, the effect of USP5 was greatly enhanced with the addition of IL-1*β*.

### 3.5. USP5 Targeted and Interacted with TRAF6

**Figure 5:**
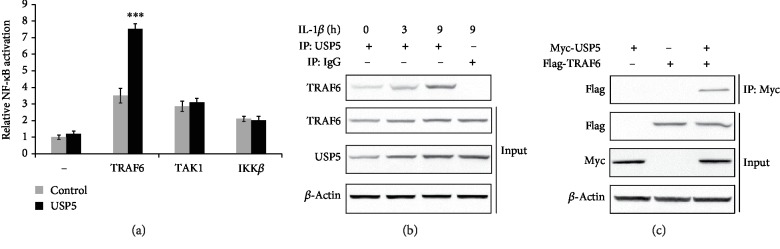
USP5 targeted and interacted with TRAF6. (a) After infection with lentivirus containing USP5 expression plasmid for 48 hours, RA-FLS were transfected with NF-*κ*B reporter plasmid and phRL-TK plasmid (internal control), together with TRAF6, TAK1, or IKK*β* expression plasmid for 24 hours. The activation of the NF-*κ*B promoter was measured by a dual-luciferase reporter gene assay. (b) Endogenous interaction between USP5 and TRAF6 was detected in RA-FLS after IL-1*β* (10 ng/ml) for indicated times. (c) Myc-USP5 and Flag-TRAF6 plasmids were transfected into RA-FLS for 48 hours; the interaction between USP5 and TRAF6 was examined. ^∗∗∗^*p* < 0.001.

By using a dual-luciferase reporter assay, we detected the effect of USP5 on NF-*κ*B activation induced by different adaptors to find the target of USP5. As shown in Figure 5(a), USP5 significantly increased TRAF6-induced NF-*κ*B activation but had no effect in the presence of TAK1 or IKK*β*. Furthermore, we observed that USP5 could interact with USP5 without IL-1*β*, and this endogenous interaction was enhanced after IL-1*β* treatment in a time-dependent manner (Figure 5(b)). Consistently, this interaction was confirmed by exogenous experiment through using plasmids with tags (Figure 5(c)).

### 3.6. USP5 Removed K48-Linked Polyubiquitination Conjugate from TRAF6

**Figure 6:**
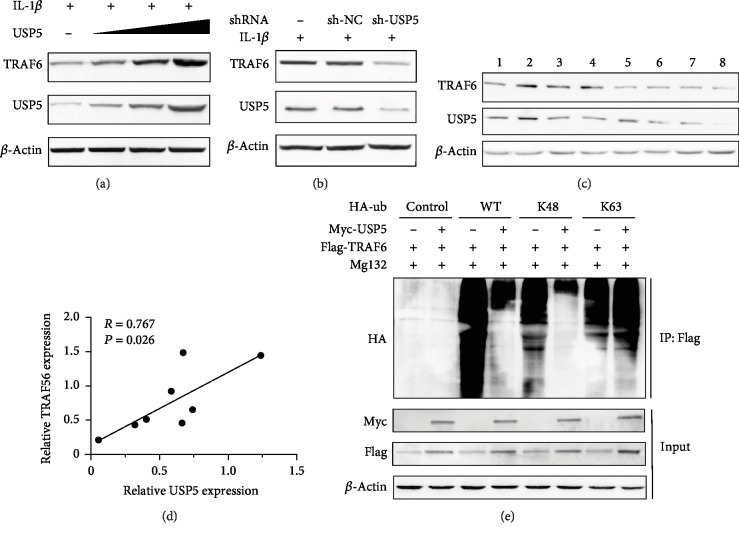
USP5 removed K48-linked polyubiquitination conjugate from TRAF6. (a) RA-FLS cells were infected with increasing titer of lentivirus that contains USP5 expression plasmid, followed by the stimulation by IL-1*β* (10 ng/ml) for 9 hours. The expression of TRAF6 was examined. (b) RA-FLS cells were infected with lentivirus that contain negative control plasmid or USP5 shRNA plasmid, followed by the stimulation by IL-1*β* (10 ng/ml) for 9 hours. The expression of TRAF6 was examined. (c) Protein levels of USP5 and TRAF6 in FLS from 8 RA patients. (d) The correlation of the relative USP5 level and relative TRAF6 level in (c) was calculated by using GraphPad Prism Software. (e) Lysates from RA-FLS cells transiently cotransfected with Myc-USP5, Flag-TRAF6, or vector control and HA-Ub (WT), HA-Ub (K48), or HA-Ub (K63) plasmids were subjected to immunoprecipitation with anti-Flag antibody followed by Western blot analysis with anti-HA antibody.

Previous studies showed that USP5 could remove K48-linked polyubiquitination chains from different substrates to affect different physiological processes [17]. However, the targets of USP5 in NF-*κ*B signaling still remain largely unknown. Therefore, we hypothesized that USP5 could affect TRAF6 expression. Indeed, we found that TRAF6 expression was greatly increased in the presence of USP5 in a dose-dependent manner (Figure 6(a)). Consistently, knockdown of the expression of USP5 led to the decrease of TRAF6 (Figure 6(b)). The expression of USP5 and TRAF6 and Pearson correlation (*R* value) in FLS from RA patients were also detected (Figures 6(c) and 6(d)). K48-linked protein polyubiquitination was well known to lead to the degradation of the corresponding protein through 26S proteasome; therefore, we performed the ubiquitination assay to examine the effect of USP5 on TRAF6 polyubiquitination. We observed that in the existence of Mg132 which is the widely used proteasome inhibitor, the wild-type polyubiquitination and K48-linked polyubiquitination levels of TRAF6 were markedly decreased in USP5 overexpressed RA-FLS cells, but the K63-linked polyubiquitination of TRAF6 was not affect by USP5 overexpression (Figure 6(e)). These data suggested that USP5 could remove K48-linked polyubiquitination conjugate from TRAF6 which therefore enhanced the expression of TRAF6 in RA-FLS.

## 4. Discussion

In the present research, we investigated the expression of USP5 in RA-FLS and explored the function of USP5 in the inflammatory process in FLS cells. To the best of our knowledge, this is the first publication which shows the effect of USP5 in RA, and for the first time, we illustrate the target of USP5 in regulating the NF-*κ*B signaling pathway.

FLS cells are the critical regulators of synovial inflammation in the progression of RA. These cells can secrete multiple proinflammatory cytokines and mediators, regulate the expression of growth factors, trigger more FLS activation as feedback, and lead to the destruction of cartilage [18]. Recently, many studies have found that FLS is a very crucial and effective treatment for RA [11, 19–21]. Therefore, to illustrate the effect of USP5 on RA, we used FLS cells in the present study, and we found that the expression of USP5 was enhanced in RA-FLS compared with OA-FLS. In addition, the USP5 level was increased upon IL-1*β* stimulation in RA-FLS, which indicated that USP5 may play important roles in the development of RA. Due to the function of secreting proinflammatory cytokines by FLS cells, we mainly detected the effect of USP5 on inflammatory responses of FLS. We found that both the mRNA and secreted protein levels of multiple proinflammatory cytokines were increased in USP5-overexpressed RA-FLS cells after IL-1*β* treatment, which suggested that USP5 could regulate the inflammation in RA-FLS upon IL-1*β* stimulation.

IL-1*β*, which is known to be correlated with much inflammatory pathology, is an important proinflammatory cytokine that regulates NF-*κ*B signaling activation [22]. NF-*κ*B has been widely reported in the pathogenesis of RA, and NF-*κ*B inhibitions have also been explored as a therapeutic approach to RA [23]. To evaluate how USP5 regulates the production of proinflammatory cytokines, we next examine the role of USP5 in NF-*κ*B activation. We found that the phosphorylation of I*κ*B*α* and p65 has a positive correlation with the USP5 expression level. In addition, dual-luciferase reporter assay results also showed that USP5-positive cell regulates the activation of NF-*κ*B.

USP5 expression was found increased in glioblastoma, melanoma, and hepatocellular carcinoma [11]. In the current study, we confirmed that the expression of USP5 is also enhanced in RA-FLS compared with that in OA-FLS. As a deubiquitinating enzyme, USP5 is always reported to be involved in regulating different physiological processes by its deubiquitination function. It has been reported that USP5 could stabilize various proteins including p53, Cav3.2, *β*-catenin, SLUG, and FoxM1 through suppressing their K48-linked polyubiquitination level [11, 12, 24, 25]; however, the target of USP5 in NF-*κ*B signaling is still unclear. In the present research, we found USP5 could interact with TRAF6 both in endogenous and exogenous conditions. Furthermore, overexpression of USP5 markedly enhanced the expression of TRAF6 in FLS. Consistently, silencing of USP5 resulted in the decreased level of TRAF6. Most importantly, we found USP5 inhibited the K48-linked polyubiquitination level of TRAF6 but did not affect its K63-linked polyubiquitination. These data suggested that USP5 removed the K48-linked polyubiquitination chains from TRAF6 and stabilized its expression, leading to the activation of NF-*κ*B and promotion of proinflammatory cytokine expression.

During our experiments, without IL-1*β* administration, we observed that USP5 could also slightly interact with TRAF6, which promote the NF-*κ*B activation and cytokine expression. However, with the exogenous IL-1*β* existence, these phenomena have been strengthened obviously. We hypothesized that it may be related to the self-production of proinflammatory cytokines by RA-FLS, even without IL-1*β* administration; the low dose of cytokines could also promote the expression of USP5 and facilitate the interaction between TRAF6 and USP5, which also indicated that USP5 is crucial for the inflammatory processes of RA-FLS.

In conclusion, our study revealed that USP5 is a positive regulator of inflammation in RA-FLS and suggested USP5 as a potential target for RA treatment.

## Figures and Tables

**Table 1:** Primers.

Number	Gene		Sequence (5′-3′)
1	USP5	F	ACC TTC GGC TTA GAC TGG GT
		R	GGA GTC TTC GTC TTC GTT GC

2	*β*-Actin	F	ATG AAG ATC CTG ACC GAG CG
		R	TAC TTG CGC TGA GGA GGA GC

3	IL-6	F	CCT TCG GTC CAG TTG CCT TCT
		R	CCA GTG CCT CTT TGC TGC TTT

4	TNF-*α*	F	CTG AAA GCA TGA TCC GGG AC
		R	CGA TCA CTC CAA AGT GCA GC

5	MMP1	F	CCT GAA GAA TGA TGG GAG GCA
		R	CTC TTG GCA AAT CTG GCG TG

## Data Availability

The original data used to support the findings of this study are available from the corresponding authors upon request.

## References

[1] McInnes I. B., Schett G. (2011). The pathogenesis of rheumatoid arthritis. *The New England Journal of Medicine*.

[2] Smolen J. S., Aletaha D., McInnes I. B. (2016). Rheumatoid arthritis. *Lancet*.

[3] Zhang Y., Yan N., Wang X., Chang Y., Wang Y. (2019). miR-129-5p regulates cell proliferation and apoptosis via IGF-1R/Src/ERK/Egr-1 pathway in RA-fibroblast-like synoviocytes. *Bioscience reports*.

[4] Li G., Xia Z., Liu Y. (2018). SIRT1 inhibits rheumatoid arthritis fibroblast-like synoviocyte aggressiveness and inflammatory response via suppressing NF-*κ*B pathway.. *Bioscience reports*.

[5] Wang Y., Jiao T., Fu W. (2019). miR-410-3p regulates proliferation and apoptosis of fibroblast-like synoviocytes by targeting YY1 in rheumatoid arthritis. *Biomedicine & pharmacotherapy*.

[6] Jiang F., Zhou H.-Y., Zhou L.-F., Wen Y. H., Gai H. H., Wu G. M. (2019). MicroRNA-421 promotes inflammatory response of fibroblast-like synoviocytes in rheumatoid arthritis by targeting SPRY1. *European Review for Medical and Pharmacological Sciences*.

[7] Wang X., Chen Z., Fan X. (2019). Inhibition of DNM1L and mitochondrial fission attenuates inflammatory response in fibroblast-like synoviocytes of rheumatoid arthritis. *Journal of cellular and molecular medicine*.

[8] Zhou L., Li L., Wang Y., Gao Q., Geng Y.-Q. (2019). Effects of RANKL on the proliferation and apoptosis of fibroblast-like synoviocytes in rheumatoid arthritis through regulating the NF-*κ*B signaling pathway. *European Review for Medical and Pharmacological Sciences*.

[9] Kong Q.-Z., Guo L.-T., Yang J.-N. (2016). Anti-inflammatory effects of TRAF-interacting protein in rheumatoid arthritis fibroblast-like synoviocytes. *Mediators of Inflammation*.

[10] Ling X., Huang Q., Xu Y. (2017). The deubiquitinating enzyme Usp5 regulates Notch and RTK signaling during Drosophila eye development. *FEBS Letters*.

[11] Li X.-Y., Wu H.-Y., Mao X.-F., Jiang L.-X., Wang Y.-X. (2017). USP5 promotes tumorigenesis and progression of pancreatic cancer by stabilizing FoxM1 protein. *Biochemical and Biophysical Research Communications*.

[12] Ma X., Qi W., Pan H., Yang F., Deng J. (2018). Overexpression of USP5 contributes to tumorigenesis in non-small cell lung cancer via the stabilization of *β*-catenin protein. *American Journal of Cancer Research*.

[13] Liu Y., Wang W.-M., Lu Y.-F. (2017). Usp5 functions as an oncogene for stimulating tumorigenesis in hepatocellular carcinoma. *Oncotarget*.

[14] Yoshioka Y., Ye Y. Q., Okada K. (2013). Ubiquitin-specific peptidase 5, a target molecule of vialinin A, is a key molecule of TNF-*α* production in RBL-2H3 cells. *PloS one*.

[15] Livak K. J., Schmittgen T. D. (2001). Analysis of Relative Gene Expression Data Using Real-Time Quantitative PCR and the 2^−ΔΔ _C_^_T_ Method. *Methods*.

[16] Lin Y., Luo Z. (2017). NLRP6 facilitates the interaction between TAB2/3 and TRIM38 in rheumatoid arthritis fibroblast-like synoviocytes. *FEBS Letters*.

[17] Xu X., Huang A., Cui X. (2019). Ubiquitin specific peptidase 5 regulates colorectal cancer cell growth by stabilizing Tu translation elongation factor. *Theranostics*.

[18] Lefèvre S., Knedla A., Tennie C. (2009). Synovial fibroblasts spread rheumatoid arthritis to unaffected joints. *Nature Medicine*.

[19] Wu J., Qu Y., Zhang Y.-P., Deng J.-X., Yu Q.-H. (2018). RHAMM induces progression of rheumatoid arthritis by enhancing the functions of fibroblast-like synoviocytes. *BMC musculoskeletal disorders*.

[20] Casnici C., Lattuada D., Crotta K. (2018). Anti-inflammatory effect of somatostatin analogue octreotide on rheumatoid arthritis synoviocytes. *Inflammation*.

[21] Wang Y., Xu N., Zhao S. (2019). miR-410-3p suppresses cytokine release from fibroblast-like synoviocytes by regulating NF-*κ*B signaling in rheumatoid arthritis. *Inflammation*.

[22] Choi Y. J., Lee W.-S., Lee E.-G., Sung M.-S., Yoo W.-H. (2014). Sulforaphane inhibits IL-1*β*-induced proliferation of rheumatoid arthritis synovial fibroblasts and the production of MMPs, COX-2, and PGE2. *Inflammation*.

[23] Chen J., Wu W., Zhang M., Chen C. (2019). Taraxasterol suppresses inflammation in IL-1*β*-induced rheumatoid arthritis fibroblast-like synoviocytes and rheumatoid arthritis progression in mice. *International Immunopharmacology*.

[24] Potu H., Peterson L. F., Pal A. (2014). Usp5 links suppression of p53 and FAS levels in melanoma to the BRAF pathway. *Oncotarget*.

[25] Meng J., Ai X., Lei Y. (2019). USP5 promotes epithelial-mesenchymal transition by stabilizing SLUG in hepatocellular carcinoma. *Theranostics*.

